# Supplementary material for: Pyruvate Dehydrogenase Kinase 1 inhibition mediated oxidative phosphorylation enhancement in cartilage promotes osteoarthritis progression
Source: BMC Musculoskelet Disord. 2023 Jul 20;24:597. doi: 10.1186/s12891-023-06585-6 (PMC10357736; doi:10.1186/s12891-023-06585-6)
Supplement: Supplementary file 1 — Additional file 1. [file 12891_2023_6585_MOESM1_ESM.zip › MF.Gsea.1653627316242/GOMF_MIRNA_BINDING.html]

Details for gene set GOMF\_MIRNA\_BINDING[GSEA]

|  || Dataset | 55457\_collapsed\_to\_symbols.cls.cls #OA\_versus\_normal.cls.cls #OA\_versus\_normal\_repos |
| Phenotype | cls.cls#OA\_versus\_normal\_repos |
| Upregulated in class | normal |
| GeneSet | GOMF\_MIRNA\_BINDING |
| Enrichment Score (ES) | -0.48669833 |
| Normalized Enrichment Score (NES) | -1.6249772 |
| Nominal p-value | 0.019197207 |
| FDR q-value | 0.14319548 |
| FWER p-Value | 0.986 |
Table: GSEA Results Summary

  

Fig 1: Enrichment plot: GOMF\_MIRNA\_BINDING      
 Profile of the Running ES Score & Positions of GeneSet Members on the Rank Ordered List

  

| SYMBOL | TITLE | RANK IN GENE LIST | RANK METRIC SCORE | RUNNING ES | CORE ENRICHMENT || 1 | RBM4 | RNA binding motif protein 4 [Source:HGNC Symbol;Acc:HGNC:9901] | 1970 | 0.364 | -0.1164 | No |
| 2 | TARBP2 | TARBP2 subunit of RISC loading complex [Source:HGNC Symbol;Acc:HGNC:11569] | 2311 | 0.325 | -0.1101 | No |
| 3 | LIN28A | lin-28 homolog A [Source:HGNC Symbol;Acc:HGNC:15986] | 3140 | 0.236 | -0.1506 | No |
| 4 | AGO1 | argonaute RISC component 1 [Source:HGNC Symbol;Acc:HGNC:3262] | 3430 | 0.208 | -0.1521 | No |
| 5 | ZC3H7A | zinc finger CCCH-type containing 7A [Source:HGNC Symbol;Acc:HGNC:30959] | 4212 | 0.136 | -0.1991 | No |
| 6 | AGO4 | argonaute RISC component 4 [Source:HGNC Symbol;Acc:HGNC:18424] | 4346 | 0.124 | -0.1969 | No |
| 7 | RBM10 | RNA binding motif protein 10 [Source:HGNC Symbol;Acc:HGNC:9896] | 6647 | -0.053 | -0.3702 | No |
| 8 | PUM2 | pumilio RNA binding family member 2 [Source:HGNC Symbol;Acc:HGNC:14958] | 7186 | -0.095 | -0.4024 | No |
| 9 | ELAVL1 | ELAV like RNA binding protein 1 [Source:HGNC Symbol;Acc:HGNC:3312] | 7426 | -0.113 | -0.4096 | No |
| 10 | FMR1 | FMRP translational regulator 1 [Source:HGNC Symbol;Acc:HGNC:3775] | 7944 | -0.151 | -0.4346 | No |
| 11 | HNRNPA2B1 | heterogeneous nuclear ribonucleoprotein A2/B1 [Source:HGNC Symbol;Acc:HGNC:5033] | 8576 | -0.200 | -0.4635 | Yes |
| 12 | TUT7 | terminal uridylyl transferase 7 [Source:HGNC Symbol;Acc:HGNC:25817] | 8629 | -0.203 | -0.4472 | Yes |
| 13 | ZC3H12A | zinc finger CCCH-type containing 12A [Source:HGNC Symbol;Acc:HGNC:26259] | 9030 | -0.234 | -0.4547 | Yes |
| 14 | SOX4 | SRY-box transcription factor 4 [Source:HGNC Symbol;Acc:HGNC:11200] | 9291 | -0.255 | -0.4492 | Yes |
| 15 | YBX1 | Y-box binding protein 1 [Source:HGNC Symbol;Acc:HGNC:8014] | 9623 | -0.285 | -0.4462 | Yes |
| 16 | PUM1 | pumilio RNA binding family member 1 [Source:HGNC Symbol;Acc:HGNC:14957] | 10145 | -0.335 | -0.4529 | Yes |
| 17 | TUT4 | terminal uridylyl transferase 4 [Source:HGNC Symbol;Acc:HGNC:28981] | 10377 | -0.359 | -0.4348 | Yes |
| 18 | POU5F1 | POU class 5 homeobox 1 [Source:HGNC Symbol;Acc:HGNC:9221] | 10898 | -0.416 | -0.4333 | Yes |
| 19 | MATR3 | matrin 3 [Source:HGNC Symbol;Acc:HGNC:6912] | 10983 | -0.428 | -0.3967 | Yes |
| 20 | DDX21 | DExD-box helicase 21 [Source:HGNC Symbol;Acc:HGNC:2744] | 11120 | -0.446 | -0.3624 | Yes |
| 21 | AGO2 | argonaute RISC catalytic component 2 [Source:HGNC Symbol;Acc:HGNC:3263] | 11246 | -0.463 | -0.3255 | Yes |
| 22 | DND1 | DND microRNA-mediated repression inhibitor 1 [Source:HGNC Symbol;Acc:HGNC:23799] | 12266 | -0.667 | -0.3375 | Yes |
| 23 | HNRNPA1 | heterogeneous nuclear ribonucleoprotein A1 [Source:HGNC Symbol;Acc:HGNC:5031] | 12356 | -0.693 | -0.2747 | Yes |
| 24 | NEAT1 | nuclear paraspeckle assembly transcript 1 [Source:HGNC Symbol;Acc:HGNC:30815] | 12374 | -0.700 | -0.2055 | Yes |
| 25 | SOX2 | SRY-box transcription factor 2 [Source:HGNC Symbol;Acc:HGNC:11195] | 12485 | -0.742 | -0.1394 | Yes |
| 26 | ZC3H7B | zinc finger CCCH-type containing 7B [Source:HGNC Symbol;Acc:HGNC:30869] | 12639 | -0.815 | -0.0692 | Yes |
| 27 | AGO3 | argonaute RISC catalytic component 3 [Source:HGNC Symbol;Acc:HGNC:18421] | 12726 | -0.888 | 0.0135 | Yes |
Table: GSEA details [plain text format]

  

Fig 2: GOMF\_MIRNA\_BINDING      
 Blue-Pink O' Gram in the Space of the Analyzed GeneSet

  

Fig 3: GOMF\_MIRNA\_BINDING: Random ES distribution      
 Gene set null distribution of ES for **GOMF\_MIRNA\_BINDING**
